# Carbaryl: MAK Value Documentation, addendum – Translation of the German version from 2024

**DOI:** 10.34865/mb6325e11_2ad

**Published:** 2026-06-30

**Authors:** Andrea Hartwig, MAK Commission

**Affiliations:** 1 Institute of Applied Biosciences, Department of Food Chemistry and Toxicology, Karlsruhe Institute of Technology (KIT) Adenauerring 20a, Building 50.41 76131 Karlsruhe Germany; 2 Permanent Senate Commission for the Investigation of Health Hazards of Chemical Compounds in the Work Area, Deutsche Forschungsgemeinschaft, Kennedyallee 40, 53175 Bonn, Germany. Further information: Permanent Senate Commission for the Investigation of Health Hazards of Chemical Compounds in the Work Area | DFG

**Keywords:** carbaryl, insecticide, pesticide, toxicity, evaluation, acetylcholinesterase inhibitor

## Abstract

Carbaryl [63-25-2] is used as an insecticide but is no longer approved in the European Union. The previous MAK value documentation and addendum do not reflect the current data situation of the substance. The MAK Commissiondecided that a new evaluation is not of high priority. The MAK value and the other classifications are therefore suspended and the substance is listed in the Section II c of the List of MAK and BAT Values for substances no longer evaluated.

**Table:** 

**MAK value**	**see Section II c of the List of MAK and BAT Values**
**Peak limitation**	**–**
	
**Absorption through the skin**	**–**
**Sensitization**	**–**
**Carcinogenicity**	**–**
**Prenatal toxicity**	**–**
**Germ cell mutagenicity**	**–**
	
**BLW (2023)**	**reduction of the acetylcholinesterase activity in erythrocytes to 70% of the reference value^a)^**
	
Synonyms	*N*-methyl-1-naphthyl carbamate
Chemical name (IUPAC)	1-naphthyl methylcarbamate
CAS number	63-25-2
Molar mass	201.22 g/mol
Melting point	145 °C (NCBI 2023)
Vapour pressure at 25 °C	1.81 × 10^–6^ hPa (NCBI 2023)
log K_OW_	2.36 (NCBI 2023)
Solubility	110 mg/l water (NCBI 2023)

^a)^ The BLW (biological guidance value) is derived as the ceiling value because of acute toxic effects.

This addendum was prepared because the previous evaluations no longer reflect the data currently available for the MAK value and for the designations and classifications of the substance.

Carbaryl is an insecticide from the class of carbamates. It inhibits acetylcholinesterase. The biological guidance value (BLW) for acetylcholinesterase inhibitors (reduction of the acetylcholinesterase activity in erythrocytes to 70% of the reference value; Lewalter 1995; Weistenhöfer et al. 2024) therefore applies to carbaryl. The BLW is derived as the ceiling value because of acute toxic effects. However, it was not investigated whether this is the most sensitive end point.

A MAK value of 5 mg/m³ I (inhalable fraction) was set in 1966. In 1972, carbaryl was designated with an “H” (for substances which can be absorbed through the skin in toxicologically relevant amounts). In 2002, the substance was assigned to Peak Limitation Category II with an excursion factor of 4 (Greim 2002, available in German only; Henschler 1973, available in German only).

Carbaryl is used as a broad-spectrum contact insecticide and pesticide and in the veterinary field against fleas in dogs and cats (Fleck 2021). Carbaryl was approved for use in the Federal Republic of Germany between 1971 and 1983, although its use was restricted in 1980 due to its harmful effect on bees. It was approved only for vines up to the five-leaf stage and after the end of flowering and only with official approval; it was banned completely in 1986. The use of carbaryl was authorized in the former German Democratic Republic until 1990 (BVL 2010). No plant protection products containing this active substance are approved in the EU (European Commission 2022 a; European Parliament and European Council 2009). Carbaryl is listed in Annex I Parts 1 and 2 of the PIC Regulation (EC) No 689/2008 (European Commission 2022 b). Exports therefore require an export notification and the express consent of the importing country. Plant protection products containing carbaryl are used in Australia and the USA (AERU 2022). No medicinal products containing carbaryl are approved in Germany (BfArM 2022).

The previous evaluations (MAK value documentation and addendum) do not reflect the currently available data. However, a re-evaluation of the substance is not a priority. Therefore, the MAK value, the peak limitation and the “H” designation have been withdrawn and carbaryl has been allocated to Section II c of the List of MAK and BAT Values (DFG 2022). This section lists substances for which the previous MAK values, designations and classifications have been withdrawn and which are no longer being reviewed at present.
